# Sorafenib enforces HIV-1 latency by selectively inhibiting PKC-driven STAT1/3 serine 727 phosphorylation

**DOI:** 10.1128/aac.00540-26

**Published:** 2026-08-03

**Authors:** Peipei Wang, Zhuoyue Meng, Jiasheng Zhou, Huixin Wu, Wei Wang, Zhiliang Gao, Ying Zhang, Jinfeng Cai

**Affiliations:** 1 Department of Infectious Diseases, The Third Affiliated Hospital of Sun Yat-sen University 144991, Guangzhou, China; 2 Institute of Human Virology, Key Laboratory of Tropical Disease Control of Ministry of Education, Zhongshan School of Medicine of Sun Yat-sen University 74644, Guangzhou, China; 3 Department of Immunology and Microbiology, Zhongshan School of Medicine of Sun Yat-sen University 74644, Guangzhou, China; 4 National Engineering Technology Research Center for Combined Vaccines, Wuhan, China; Chinese Academy of Medical Sciences & Peking Union Medical College, Beijing, China

**Keywords:** HIV-1, sorafenib, latency-promoting agent, STAT1/3 Ser727

## Abstract

Eliminating the latent HIV-1 reservoir remains a major barrier to achieving a functional cure. The “block-and-lock” strategy aims to enforce durable transcriptional silencing of proviruses, thereby preventing viral rebound upon interruption of antiretroviral therapy. However, the molecular mechanisms governing stable latency remain incompletely understood. Here, we identify protein kinase C (PKC)-dependent phosphorylation of STAT1 and STAT3 at serine 727 as a previously unrecognized regulatory checkpoint controlling HIV-1 transcriptional reactivation. Pharmacological inhibition of this axis using sorafenib, a clinically approved multi-kinase inhibitor, robustly suppresses HIV-1 reactivation across multiple latency models and promotes the establishment of latency in primary CD4^+^ T cells. Mechanistically, sorafenib attenuates phorbol 12-myristate 13-acetate (PMA)-induced activation of the JAK-STAT and PI3K-Akt signaling pathways, and selectively blocks PKC-mediated phosphorylation of STAT1/3 at serine 727 without affecting canonical tyrosine phosphorylation (Y701/Y705). This selective inhibition disrupts transcriptional activation at the HIV-1 long terminal repeat (*LTR*), thereby preventing viral reactivation. Importantly, sorafenib does not induce detectable cytotoxicity or alter activation and exhaustion markers in primary CD4^+^ T cells, supporting a favorable safety profile *in vitro*. Collectively, our findings define a critical role for STAT1/3 Ser727 in the regulation of HIV-1 latency and establish sorafenib as a promising latency-promoting agent for future applications in the development of a functional cure for HIV-1.

## INTRODUCTION

Acquired immunodeficiency syndrome (AIDS), caused by infection with human immunodeficiency virus type 1 (HIV-1), remains a formidable global health challenge, affecting approximately 39.9 million individuals worldwide and contributing to over 1.3 million new infections annually (1). Although antiretroviral therapy (ART) has revolutionized HIV-1 management by transforming it from a fatal disease into a chronic but controllable condition, it is incapable of eliminating the latent viral reservoir within CD4^+^ T cells (2, 3). These transcriptionally silent reservoirs persist by evading both immune surveillance and ART, thereby posing a major obstacle to viral eradication (4–6). As a result, lifelong ART is required, which is associated with cumulative drug toxicity, the emergence of drug-resistant viral strains, and substantial socioeconomic burdens. Therefore, achieving a functional HIV-1 cure necessitates either complete eradication of the latent reservoir or its permanent silencing.

Hematopoietic stem cell transplantation using CCR5Δ32/Δ32 donor cells represents a promising strategy for HIV-1 cure by effectively blocking viral entry and replacing susceptible immune cells (7, 8). However, its clinical application is severely limited by the scarcity of compatible donors, the risk of graft-versus-host disease, and transplant-related mortality, restricting its use primarily to patients with concurrent hematologic malignancies (9, 10). Immunotherapeutic approaches, including broadly neutralizing antibodies (bNAbs) and chimeric antigen receptor T cell therapy, have also shown considerable potential in HIV-1 eradication. Nevertheless, their efficacy remains constrained by antigenic escape, an immunosuppressive microenvironment, and the requirement for sustained immune pressure (11–13). Gene-editing technologies, particularly CRISPR/Cas9-based approaches, offer transformative potential by directly excising or inactivating proviral DNA. However, challenges such as viral genetic diversity, escape mutations, and the risk of off-target effects continue to impede their clinical translation (14–18). The “shock and kill” strategy remains a major focus of HIV-1 cure research, aiming to eliminate latent reservoirs through pharmacological reactivation of dormant proviruses followed by immune-mediated clearance (19, 20). However, this approach is limited by the suboptimal potency and safety of latency-reversing agents (LRAs), insufficient immune clearance, and the complex heterogeneity of latent reservoirs (21–23).

While prevailing HIV-1 cure strategies focus on reactivating and eliminating latent viral reservoirs, an alternative “block and lock” strategy attempts to “block” HIV-1 latent reservoirs by deepening the latency of the HIV-1 provirus and continuously “lock” the expression of provirus until the natural death of host cells, thus allowing the safe withdrawal of the ART. Drugs with a “block and lock” function are called latency-promoting agents (LPAs) (24–26). Existing LPAs can be systematically classified based on their mechanisms of action: (i) Tat-targeting agents, including didehydro-cortistatin A (dCA) (27–29), Triptolide (30), and Q308 (31). (ii) Host factor-targeting inhibitors, such as AUY922 and 17-AAG (32, 33), ruxolitinib and tofacitinib (34, 35), ponatinib (36), and ripretinib (37). (iii) Epigenetic modulators, including spironolactone (38, 39), decitabine (40), and wogonin (41). Although several LPAs have demonstrated the ability to induce suboptimal viral transcription and hinder reactivation, none have yet achieved the desired clinical efficacy (24–26). Therefore, the identification of novel and more potent LPAs remains crucial in advancing the “block and lock” strategy as a viable pathway toward a functional cure for HIV-1.

In this study, we performed high-throughput screening of an epigenetics-focused compound library comprising 851 molecules using J-Lat A2 cells and identified Sorafenib as a potent inhibitor of latent HIV-1 reactivation. Mechanistically, sorafenib selectively blocks protein kinase C (PKC)-mediated phosphorylation of STAT1 and STAT3 at serine 727 within their transcriptional activation domains (TADs), thereby suppressing HIV-1 transcriptional reactivation. These findings provide a mechanistic basis for the latency-promoting activity of sorafenib and highlight its potential as a candidate LPA for the development of therapeutic strategies aimed at achieving a functional cure for HIV-1.

## RESULTS

### High-throughput screening identified sorafenib as a potential LPA candidate

**Fig 1:**
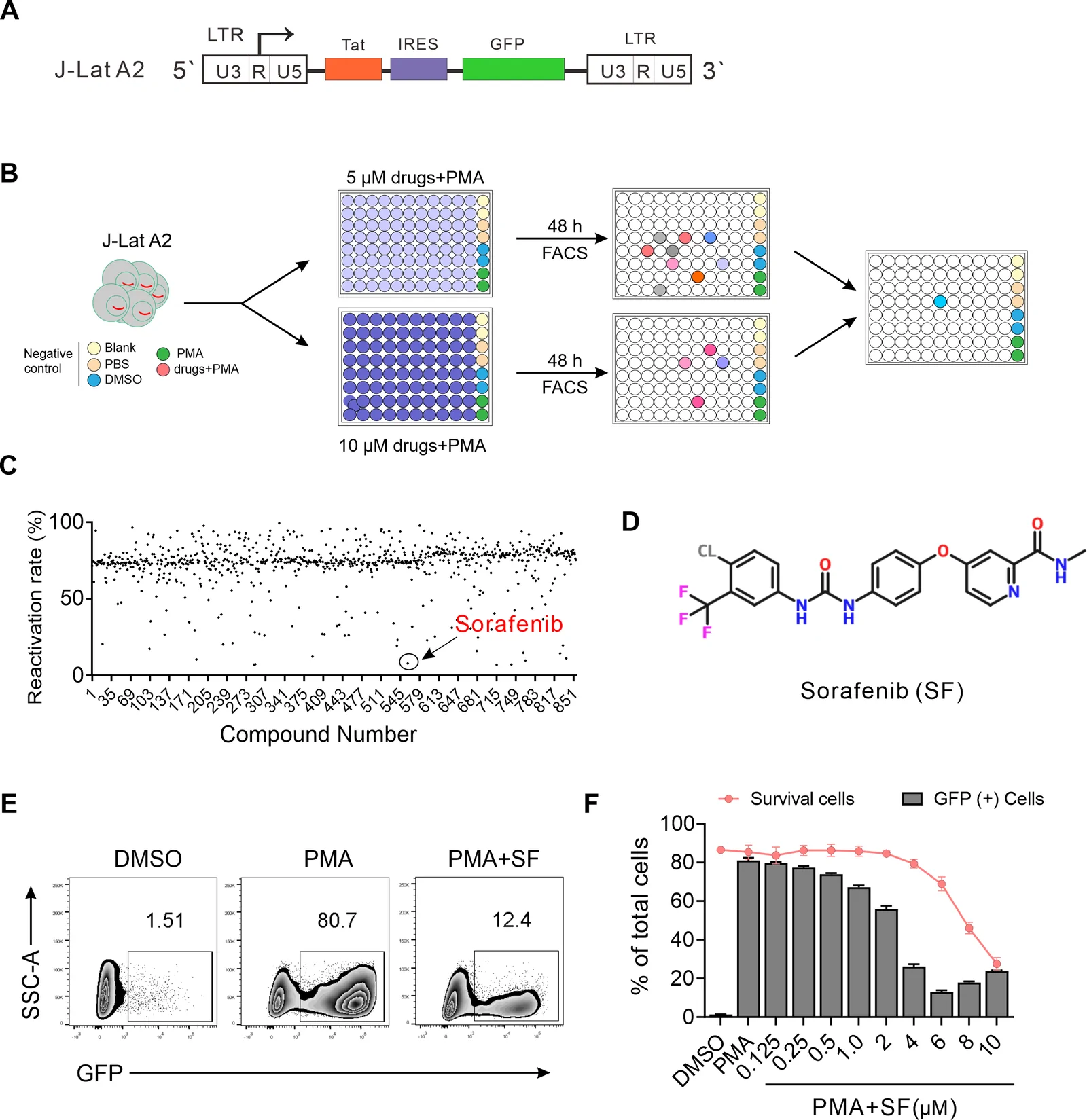
Screening of an epigenetics-focused compound library identified sorafenib as a suppressor of latent HIV-1 reactivation. (**A**) Diagram of integrated HIV-1 genomic structure in J-Lat A2 cells. (**B**) The process of high-throughput screening in the J-Lat A2 cell line. J-Lat A2 cells were treated with 10 μM or 5 μM drugs plus PMA for 48 h. Furthermore, the blocking efficiency was measured according to the percentage of GFP^+^ cells. (**C**) In the summary of screening results, reactivation efficiency was measured by flow cytometry as the percentage of GFP^+^ cells. (**D**) Chemical structure of sorafenib. (**E**) Representative flow cytometry plots of sorafenib-mediated suppression in J-Lat A2 cells. (**F**) Cell viability and reactivation of latent HIV-1 were measured after the indicated treatment. J-Lat A2 cells were co-treated with sorafenib at the indicated concentrations and PMA (10 ng/mL). The percentage of GFP^+^ cells and cell viability were plotted. Data are means ± SD for three technical replicates from one representative experiment of three independent experiments.

To identify novel LPAs, we performed a high-throughput screening of a commercially available epigenetics-focused compound library comprising 851 bioactive modulators targeting chromatin regulators, histone-modifying enzymes, and DNA methylation pathways. Screening was conducted in J-Lat A2 T cells, a well-established model of HIV-1 latency widely used to study proviral transcriptional regulation (42). This model contains an integrated HIV-1 long terminal repeat (LTR)-driven Tat-GFP reporter, enabling quantitative assessment of proviral reactivation at a defined genomic integration site (Fig. 1A). Under basal conditions, GFP expression remained low (1%–3%), consistent with transcriptional silencing of the provirus. In contrast, stimulation with phorbol 12-myristate 13-acetate (PMA, 10 ng/mL), a potent PKC activator, robustly induced HIV-1 reactivation, increasing GFP^+^ cells to approximately 80% within 48 h.

We then screened compounds at 5 μM and 10 μM under PMA-induced reactivation conditions to identify agents capable of suppressing proviral transcription (Fig. 1B). This primary screen yielded more than 80 candidate hits (Fig. 1C), which were subsequently subjected to secondary validation across multiple concentrations. To prioritize clinically relevant candidates, we further integrated pharmacological target information, focusing on compounds acting on key signaling nodes, including Janus kinases, platelet-derived growth factor receptors (PDGFR), vascular endothelial growth factor receptors (VEGFR), and RAF kinases. Together with considerations of efficacy, toxicity, and translational feasibility, these analyses identified sorafenib as a top candidate for further investigation.

Sorafenib is an orally available multikinase inhibitor targeting RAF, VEGFR, and PDGFR signaling pathways and is clinically approved for the treatment of advanced renal cell carcinoma and hepatocellular carcinoma (Fig. 1D). Notably, its role in HIV-1 latency regulation has not been previously explored. Validation experiments confirmed that sorafenib significantly suppresses PMA-induced HIV-1 reactivation in J-Lat A2 cells (Fig. 1E), exhibiting a clear dose-dependent inhibitory effect (Fig. 1F). The optimal concentration for maximal suppression under PMA stimulation was determined to be 4 μM over 48 h (Fig. 1F). The reduced inhibitory activity at higher concentrations (8 and 10 μM) is likely attributable to cytotoxicity (Fig. 1F), which confounds the GFP^+^ cell percentage through selective cell loss. Crucially, sorafenib effectively suppressed GFP^+^ cells without compromising viability at concentrations ≤6 µM, supporting its potential as a latency-promoting agent.

### Sorafenib exhibits broad-spectrum suppression of latent HIV-1 reactivation

**Fig 2:**
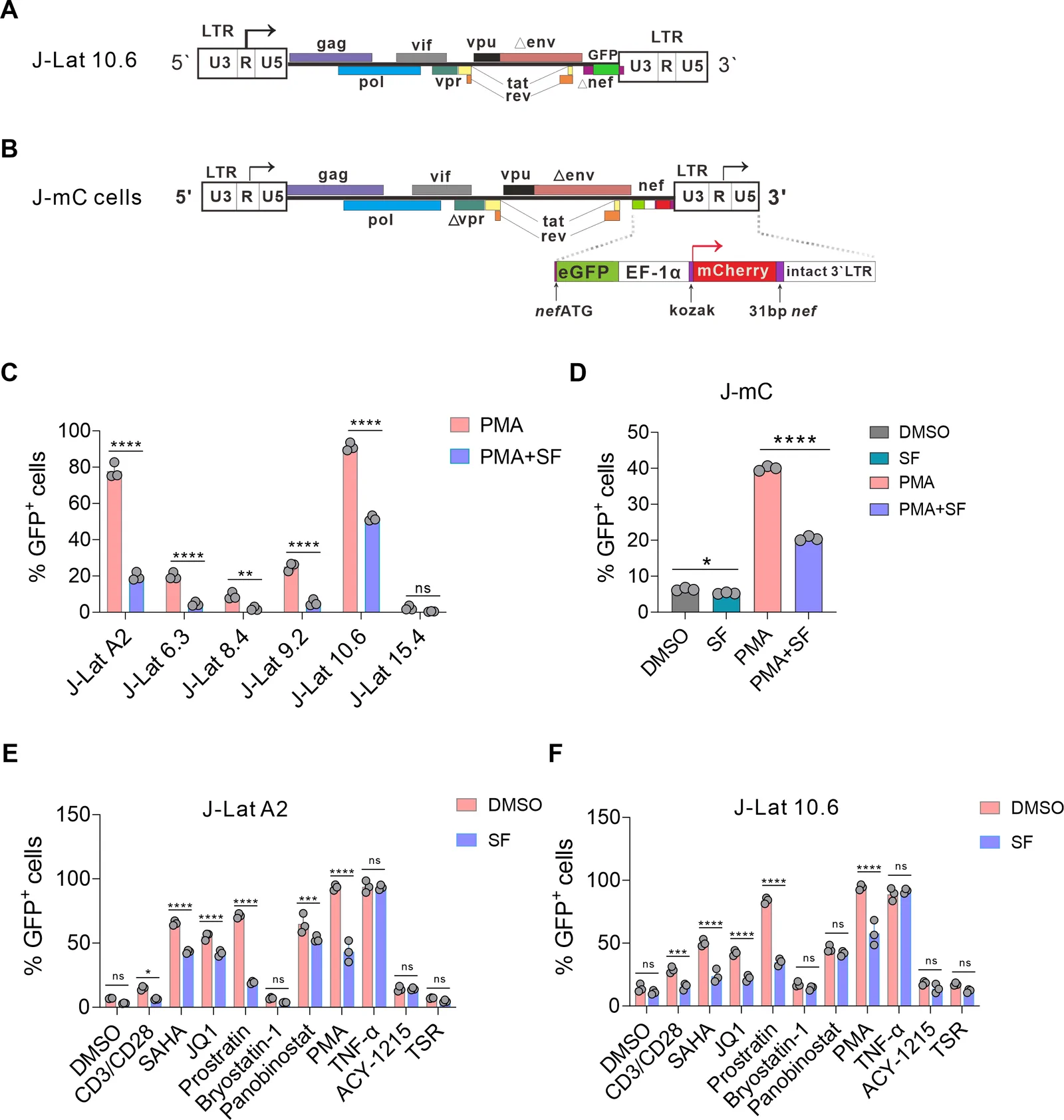
Sorafenib exhibits a broad-spectrum inhibitory effect on latent HIV-1 reactivation. (**A**) Diagram of integrated HIV-1 genomic structure in J-Lat 10.6 cells. (**B**) Diagram of integrated HIV-1 genomic structure in J-mC cells. (**C**) The inhibitory effect of sorafenib on HIV-1 latency reactivation in J-Lat cell lines with different integrated sites. (**D**) The inhibitory effect of sorafenib on HIV-1 latency reactivation in J-mC cells. (**E and F**) The inhibitory effect of sorafenib on different LRAs-induced HIV-1 reactivation in J-Lat A2 cells (**E**) and J-Lat 10.6 cells (**F**). Anti-CD3 antibody (1 μg/mL), anti-CD28 antibody (1 μg/mL), SAHA (1 μM), JQ1 (1 μM), prostratin (1 μM), bryostatin-1 (1 μM), panobinostat (10 nM), PMA (10 ng/mL), TNF-α (10 ng/mL), ACY-1215 (3 μM), thiostrepton (TSR; 1 μM), and sorafenib (4 μM) were used. Each group of data represents the average of three replicates. All data are means ± SD. *P* values calculated using unpaired *t*-test, ns, not significant, *, *P* < 0.05, **, *P* < 0.01, ***, *P* < 0.001, and ****, *P* < 0.0001. Brackets indicating specific group comparisons are shown on each graph.

The establishment of HIV-1 latency is highly heterogeneous, largely influenced by the genomic integration site and local chromatin environment, posing a major barrier to achieving a functional cure. To determine whether sorafenib exerts a broad and integration site-independent effect on latent HIV-1 reactivation, we evaluated its activity across multiple J-Lat clonal cell lines, including J-Lat 6.3, 8.4, 9.2, 10.6, and 15.4. These clones, derived from Jurkat cells infected with the same reporter virus, harbor distinct proviral integration sites within the host genome (Fig. 2A and Table S1).

Treatment with sorafenib (4 μM) robustly suppressed PMA-induced HIV-1 reactivation across all J-Lat clones tested, with the exception of J-Lat 15.4 (Fig. 2C). The insensitivity of J-Lat 15.4 to sorafenib may reflect a particularly repressive chromatin context at its specific integration site, which may already be deeply silenced and less dependent on the PKC-STAT signaling axis targeted by sorafenib. Consistently, sorafenib also significantly inhibited viral reactivation in the J-mC stable latency model, which contains a full-length HIV-1 genome with dual fluorescent reporters and diverse integration contexts (Fig. 2B and D) (43). Together, these findings indicate that the inhibitory effect of sorafenib is largely independent of proviral integration site variability. Given that LRAs activate HIV-1 transcription through distinct upstream signaling pathways (44), we next assessed whether sorafenib could counteract reactivation induced by mechanistically diverse stimuli. In both J-Lat A2 and J-Lat 10.6 cells, sorafenib markedly suppressed HIV-1 reactivation triggered by multiple LRAs, including vorinostat (SAHA), JQ1, prostratin, and anti-CD3/CD28 stimulation (Fig. 2E and F). Collectively, these data demonstrate that sorafenib exerts a broad-spectrum inhibitory effect on HIV-1 reactivation across multiple latency models, irrespective of proviral integration site or upstream activating stimuli, suggesting the involvement of a convergent host regulatory mechanism controlling viral transcriptional reactivation.

### Sorafenib can inhibit HIV-1 transcription and promote HIV-1 latency in primary CD4^+^ T cells

**Fig 3:**
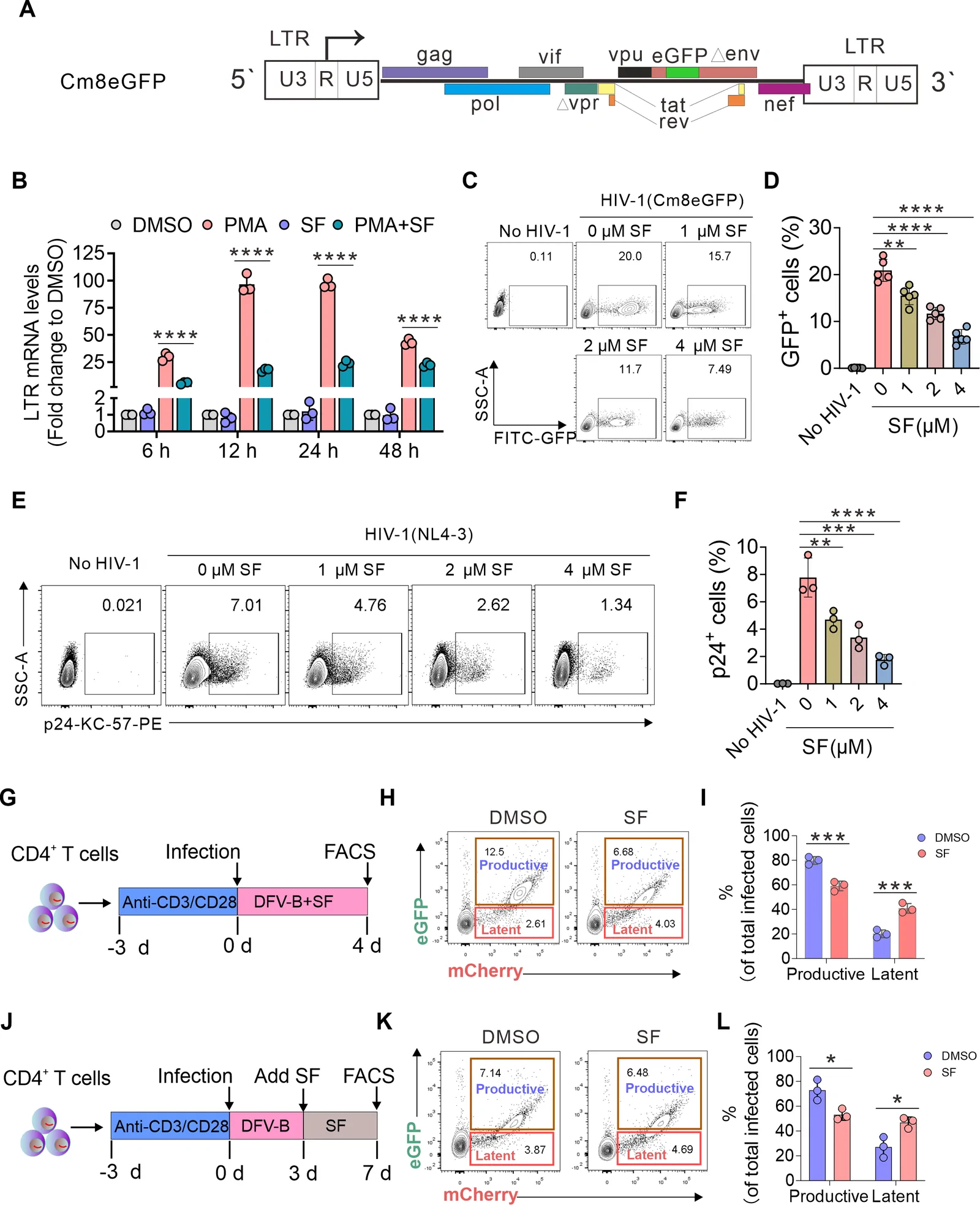
Sorafenib promotes HIV-1 latency in primary CD4^+^ T cells. (**A**) Schematic diagram of the Cm8eGFP viral genome. (**B**) HIV-1 transcription level with indicated treatment in primary CD4^+^ T cells under HIV-1 infection. (**C and D**) Sorafenib inhibits Cm8eGFP infection in primary CD4^+^ T cells. Primary CD4^+^ T cells were isolated from healthy donors and then were activated with anti-CD3/anti-CD28 antibodies for 3 days. Activated primary CD4^+^ T cells were infected with Cm8eGFP in medium containing sorafenib. (**C**) Flow cytometric chart of Cm8eGFP-infected primary CD4^+^ T cells on day 3 post-infection. (**D**) Proportion of HIV-1-infected (GFP^+^) cells. (**E–F**) Sorafenib inhibits live virus (NL4-3) infection in primary CD4^+^ T cells. (**E**) Flow cytometric chart of NL4-3 infected primary CD4^+^ T cells on day 3 post-infection. (**F**) Proportion of HIV-1-infected (HIV-1 p24^+^) cells. (**G through I**) Sorafenib inhibits productive HIV-1 infection and promotes the percentage of latently infected cells. (**G**) Activated primary CD4^+^ T cells were infected with DFV-B in medium containing sorafenib. Productively infected cells and latently infected cells were detected on day 4 post-infection. (**H**) Flow cytometric chart of DFV-B-infected primary CD4^+^ T cells on day 4 post-infection. Productively infected cells were identified as mCherry^+^GFP^+^ and latently infected cells as mCherry^+^GFP⁻. (**I**) Proportion of productively infected cells and latently infected cells in total infected cells on day 4 post-infection. (**J–L**) Sorafenib increases the proportion of latently infected cells in total infected cells on day 7 post-infection. (**J**) Scheme of experimental procedure. Sorafenib (4 μM) was added on day 3 post-infection and maintained continuously until day 7. (**K**) Representative flow cytometry plots on day 7 post-infection. (**L**) Proportion of productively infected cells and latently infected cells in total infected cells on day 7 post-infection. Data are means ± SD for three biological replicates from independent donors. *p*-values were calculated using two-way ANOVA (or mixed model). *, *P* < 0.05, **, *P* < 0.01, ***, *P* < 0.001, and ****, *P* < 0.0001.

Building on our findings in cell line models, we next investigated whether sorafenib exerts similar effects in primary human CD4^+^ T cells. Activated CD4^+^ T cells from healthy donors were infected with a single-round fluorescent HIV-1 reporter virus (Cm8eGFP), in which the *env* gene is replaced with an eGFP reporter cassette, the virus is pseudotyped with CXCR4-tropic envelope and is replication-incompetent, allowing quantification of *LTR*-driven transcription without the confounding effects of viral spread (Fig. 3A). Viral transcriptional activity was assessed by measuring HIV-1 *LTR*-derived mRNA at multiple time points post-infection (4, 12, 24, and 48 h), as well as viral protein expression by flow cytometry at 48 h. Sorafenib treatment significantly reduced both viral transcription and protein expression in a dose-dependent manner (Fig. 3A through C), indicating robust suppression of early HIV-1 gene expression in primary cells. To further validate the inhibitory effect of sorafenib in a replication-competent HIV-1 infection model, we performed additional live virus experiments. Consistent with the results obtained using the Cm8eGFP single-round reporter system, sorafenib significantly suppressed HIV-1 transcription (Fig. 3E and F), further supporting its potential role as a latency-promoting agent.

To directly assess whether sorafenib influences the fate of infected cells, we employed a dual-fluorescent reporter system (DFV-B) that enables simultaneous discrimination between productively infected and latently infected cells (42). In this system, productively infected cells were identified as mCherry^+^GFP^+^, reflecting active HIV-1 *LTR*-driven transcription, while latently infected cells were defined as mCherry^+^GFP⁻, indicating successful viral integration (marked by *EF-1α*-driven mCherry expression) with transcriptionally silent *LTR* (absence of GFP). Uninfected cells were gated as mCherry⁻GFP⁻. Activated CD4^+^ T cells were infected in the presence or absence of sorafenib, and infection outcomes were analyzed by flow cytometry. As shown in Fig. 3G through I, sorafenib markedly reduced the proportion of productively infected cells while concomitantly increasing the fraction of latently infected cells within the infected population, indicating a shift in infection fate toward latency establishment. To further determine whether this effect persists after viral integration, sorafenib was added in a delayed treatment setting following initial infection (day 3 post-infection), when proviral integration had already occurred. Sorafenib (4 μM) was maintained continuously in the culture medium from day 3 until the analysis endpoint at day 7 post-infection. Notably, delayed exposure to sorafenib still significantly increased the proportion of latently infected cells among infected populations (Fig. 3J through L), suggesting that sorafenib can reinforce or stabilize latency even after early stages of infection. Collectively, these results demonstrate that sorafenib suppresses HIV-1 transcription and promotes a shift from productive infection toward latent infection in primary CD4^+^ T cells, both during early infection and after proviral integration, supporting its role as a potent latency-promoting agent.

### Sorafenib does not induce significant cytotoxicity, cell activation, or cell dysfunction

**Fig 4:**
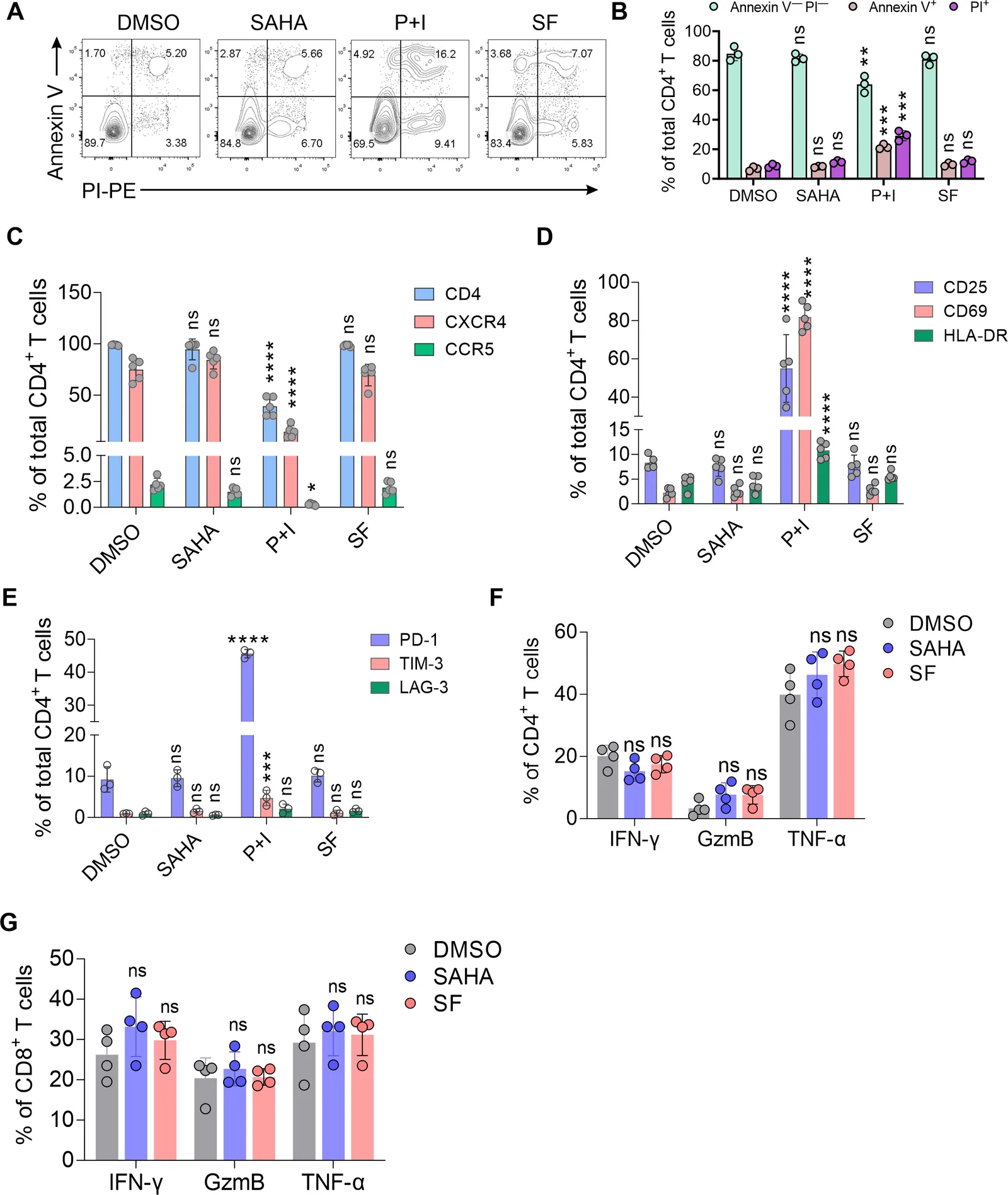
Sorafenib does not cause apparent cytotoxicity, cell activation, or cell dysfunction. (**A**) Unactivated primary CD4^+^ T cells were incubated with DMSO, PMA (10 ng/mL) plus ionomycin (1 μM), SAHA (1 μM), or sorafenib (4 μM) for 48 h and then analyzed for apoptosis using annexin V/PI staining assays. (**B**) Data from panel A are displayed graphically, including proportions of live cells (Annexin V⁻/PI⁻), early apoptotic, late apoptotic, and primary necrosis. (**C**) Expression of HIV-1 coreceptors CXCR4 or CCR5 on CD4^+^ T cells following sorafenib treatment. (**D**) Expression of activation markers CD25, CD69, or HLA-DR on CD4^+^ T cells following sorafenib treatment. (**E**) Expression of exhaustion markers PD-1, TIM-3, or LAG-3 on CD4^+^ T cells following sorafenib treatment. Compound concentrations were as follows: SAHA (1 μM), PMA (10 ng/mL), ionomycin (1 μM), and sorafenib (4 μM). (**F and G**) Effects of sorafenib on the cytokine secretion of CD4^+^ T cells (**F**) and CD8^+^ T cells (**G**). Unactivated CD4^+^ T cells or CD8^+^ T cells isolated from HIV-1-naive individuals were exposed to the indicated treatment for 48 h. Cells were then washed and cultured in fresh medium with PMA and ionomycin for 4 h in the presence of brefeldin A. IFN-γ, GzmB, or TNF-α production in CD4^+^ T cells or CD8^+^ T cells was measured by intracellular cytokine staining. Each group of data represents the average of three replicates. All data are means ± SD. *P*-values were calculated using an unpaired *t*-test, ns, not significant, *, *P* < 0.05, ***, *P* < 0.001, and ****, *P* < 0.0001. Brackets indicating specific group comparisons are shown on each graph.

To evaluate the safety profile of sorafenib as an LPA, we first assessed its cytotoxicity in primary CD4^+^ T cells. Unactivated CD4^+^ T cells were treated with DMSO, SAHA, PMA plus ionomycin, or sorafenib (4 μM) for 48 h. Cell viability was analyzed by Annexin V/propidium iodide (PI) staining. The proportion of live cells (Annexin V⁻/PI⁻) was quantified to evaluate cytotoxicity, and no detectable cytotoxic effects of sorafenib were observed under the tested conditions (Fig. 4A and B). To determine whether sorafenib alters HIV-1 susceptibility, we next examined the expression of key viral entry receptors, including CD4, CCR5, and CXCR4. Flow cytometric analysis showed that sorafenib treatment did not significantly affect the surface expression of these receptors, indicating that it does not modify cellular susceptibility to HIV-1 infection (Fig. 4C). We further evaluated whether sorafenib induces T cell activation. In contrast to PMA plus ionomycin, sorafenib did not upregulate canonical activation markers, including CD25, CD69, or HLA-DR, suggesting that it does not trigger global T cell activation or inflammatory activation programs (Fig. 4D). In addition, we assessed markers of T cell exhaustion and effector dysfunction. Sorafenib treatment did not increase the expression of exhaustion-associated markers in CD4^+^ T cells (Fig. 4E), nor did it induce significant production of effector cytokines, including IFN-γ, TNF-α, or granzyme B, in either CD4^+^ or CD8^+^ T cells derived from the same donors (Fig. 4F and G). Collectively, these data demonstrate that sorafenib does not compromise viability, activation state, or functional integrity of primary T cells, supporting a favorable safety profile and reinforcing its suitability as a candidate latency-promoting agent for HIV-1 “block-and-lock” strategies.

### Sorafenib suppresses latent HIV-1 reactivation through selective inhibition of STAT1/3 serine 727 phosphorylation

**Fig 5:**
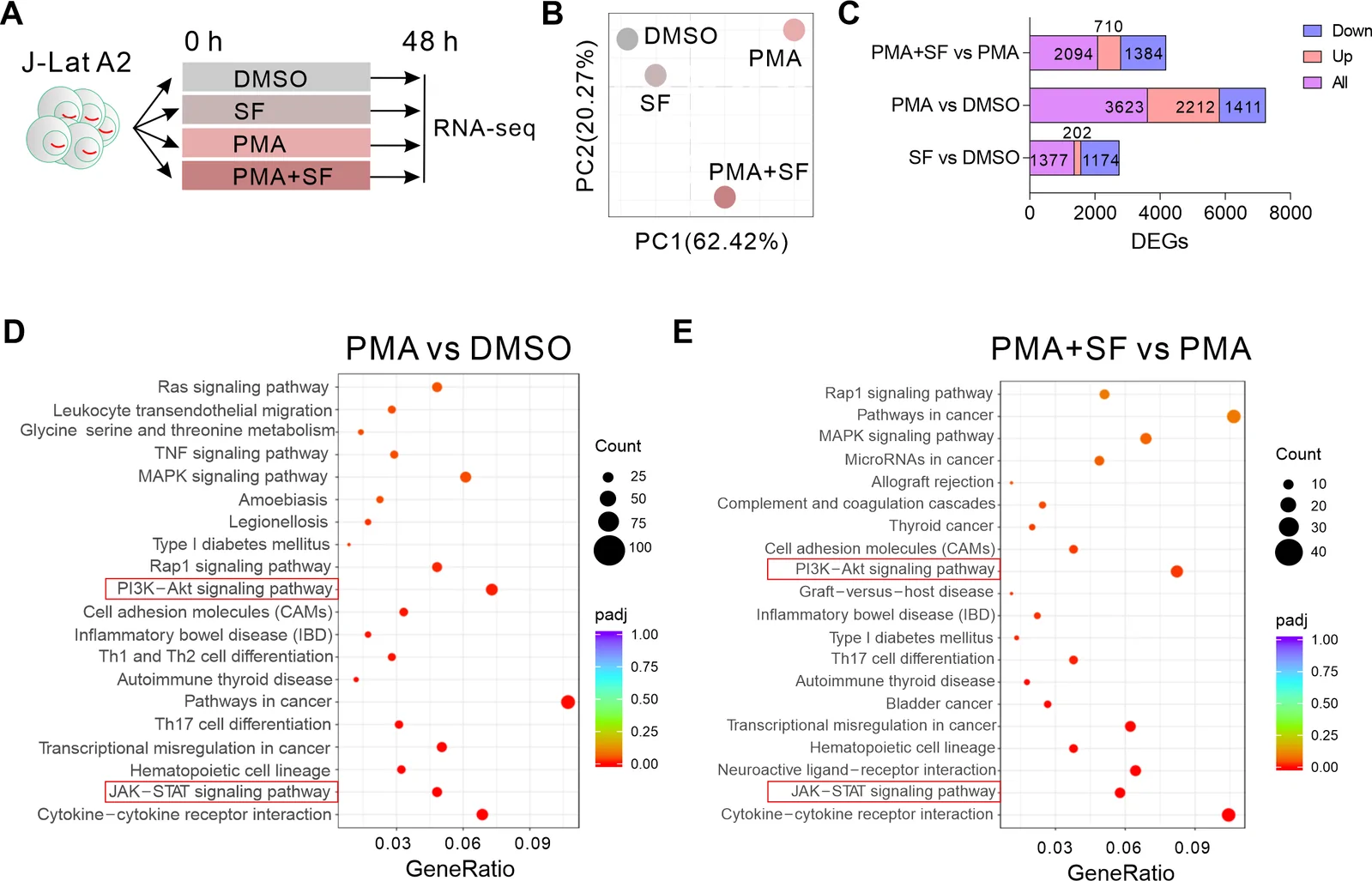
Sorafenib suppresses activation of JAK-STAT and PI3K-Akt signaling pathways. (**A**) RNA-seq experimental design and workflow. J-Lat A2 cells were incubated with DMSO, PMA (10 ng/mL), sorafenib (4 μM), and PMA (10 ng/mL) plus sorafenib (4 μM) for 48 h and then collected for RNA-seq. (**B**) Principal component analysis (PCA) of transcriptomic profiles across treatment groups. (**C**) Statistical comparison of differentially expressed genes (DEGs) between sample groups. (**D and E**) KEGG pathway enrichment analysis of DEGs in PMA vs DMSO (**D**) or PMA + SF vs PMA (**E**). Red boxes highlight the JAK-STAT and PI3K-Akt signaling pathways.

To elucidate the molecular mechanism underlying sorafenib-mediated suppression of latent HIV-1 reactivation, we first performed transcriptomic profiling of J-Lat A2 cells treated with DMSO, sorafenib, PMA, or PMA in combination with sorafenib for 48 h (Fig. 5A). Principal component analysis revealed clearly segregated transcriptional signatures among treatment groups, with PMA-treated cells forming a distinct cluster that was substantially reversed by sorafenib (Fig. 5B). Differential gene expression analysis further demonstrated that PMA induced broad transcriptional activation, whereas sorafenib effectively attenuated these PMA-driven transcriptional programs (Fig. 5C). Kyoto Encyclopedia of Genes and Genomes (KEGG) pathway enrichment analysis revealed that sorafenib significantly counteracted PMA-induced activation of signaling pathways associated with JAK-STAT and PI3K-Akt signaling (Fig. 5D and E). These results suggested that sorafenib suppresses latent HIV-1 reactivation by modulating upstream signaling events that converge on transcriptional regulation of the viral LTR.

**Fig 6:**
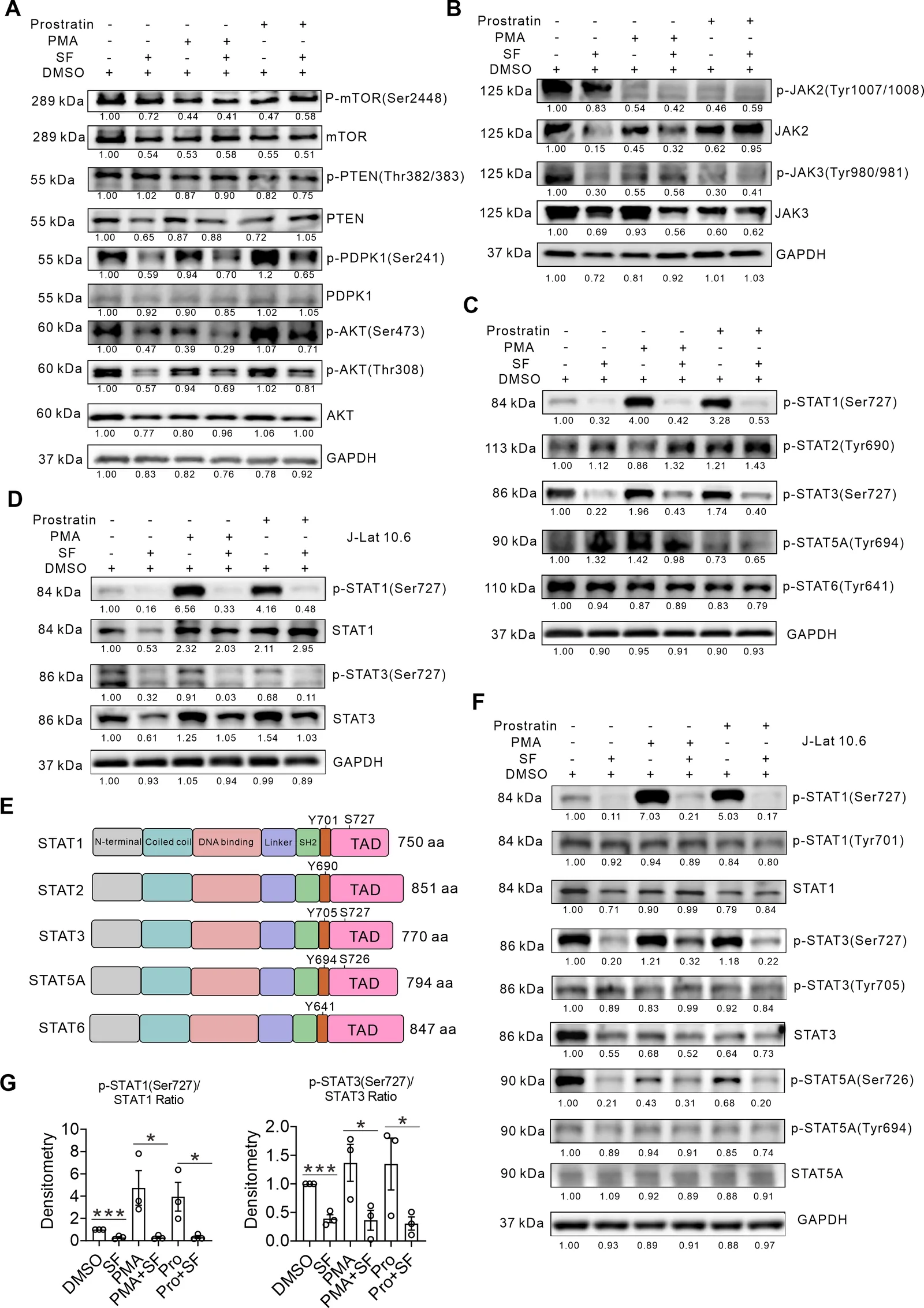
Sorafenib suppresses latent HIV-1 reactivation by inhibiting serine 727 phosphorylation in the STAT family. (**A**) Effects of sorafenib on expression and phosphorylation of core proteins in the PI3K-AKT-mTOR signaling pathway. J-Lat A2 cells were treated with DMSO, sorafenib (4 μM), PMA (10 ng/mL), prostratin (4 μM), PMA plus sorafenib, or prostratin plus sorafenib for 48 h and then collected for western blot. (**B**) Effects of sorafenib on expression and phosphorylation of JAK2 and JAK3 in J-Lat A2 cells. (**C**) Effects of sorafenib on phosphorylation levels across STAT family proteins in J-Lat A2 cells. (**D**) Effects of sorafenib on expression and phosphorylation of STAT1 and STAT3 in J-Lat A2 cells. (**E**) Structural domains and phosphorylation sites of STAT proteins. STATs (750–900 amino acids) comprise six domains: N-terminal, coiled-coil, DNA-binding, linker, SH2, and transcriptional activation domain (TAD). Phosphorylation sites: “Y” (tyrosine) and “S” (serine). (**F**) Effects of sorafenib on expression and phosphorylation of STAT1, STAT3, and STAT5 in J-Lat 10.6 cells. Densitometric semi-quantification of bands normalized to the untreated control is shown below the immunoblot bands. (**G**) Densitometric quantification of p-STAT1 (Ser727)/STAT1 and p-STAT3 (Ser727)/STAT3 ratios based on western blot analyses. Data are presented as mean ± SD from three independent experiments. *P*-values calculated using unpaired *t*-test, *P < 0.05 and ***P < 0.001.

To identify the specific signaling nodes responsible for this effect, we next systematically examined the phosphorylation status of key proteins within the PI3K-AKT-mTOR and JAK-STAT pathways. J-Lat A2 cells were treated with DMSO, sorafenib, PMA, PMA plus sorafenib, prostratin, or prostratin plus sorafenib, followed by western blot analysis. Sorafenib had no appreciable effect on the phosphorylation of mTOR, PTEN, or STAT family members at canonical tyrosine residues, including STAT2 (Y690), STAT5 (Y694), and STAT6 (Y641). In contrast, sorafenib markedly reduced phosphorylation of PDPK1, AKT, JAK2, and JAK3, indicating broad modulation of upstream signaling cascades activated by PMA stimulation. Importantly, despite these upstream effects, the most consistent and pronounced inhibition was observed at the level of STAT1 and STAT3 serine 727 phosphorylation (Fig. 6A through C). To further validate this observation, we examined an independent J-Lat model (J-Lat 10.6), which confirmed that sorafenib selectively suppressed serine 727 phosphorylation of STAT1 and STAT3, while leaving canonical tyrosine phosphorylation events unaffected (Fig. 6D). Notably, sorafenib also reduced total STAT1 and STAT3 protein levels in J-Lat 10.6 cells (Fig. 6D), suggesting effects beyond direct inhibition of serine 727 phosphorylation. This reduction may involve post-transcriptional regulatory mechanisms, including altered protein turnover or mRNA stability. Reduced total STAT1/3 levels may further contribute to the latency-promoting effects of sorafenib by limiting the pool of STAT proteins available for activation. Future studies will be needed to further clarify the underlying mechanisms. Structurally, STAT proteins contain conserved domains including an N-terminal domain, coiled-coil domain, DNA-binding domain, linker domain, SH2 domain, and a TAD (Fig. 6E). While tyrosine phosphorylation is conserved across STAT family members and is required for dimerization and nuclear translocation, serine phosphorylation within the TAD is more selectively distributed and is known to fine-tune transcriptional activity. Based on this structural distinction, we hypothesized that sorafenib preferentially targets STAT-mediated transcriptional activation through modulation of TAD-associated serine phosphorylation. Consistently, integrated analysis across STAT family members confirmed that sorafenib selectively suppresses serine phosphorylation (Fig. 6F and G). These findings demonstrate that sorafenib enforces viral silencing by selectively abrogating PKC-induced phosphorylation of STAT1 and STAT3 at serine 727, thereby preventing latent HIV-1 reactivation.

**Fig 7:**
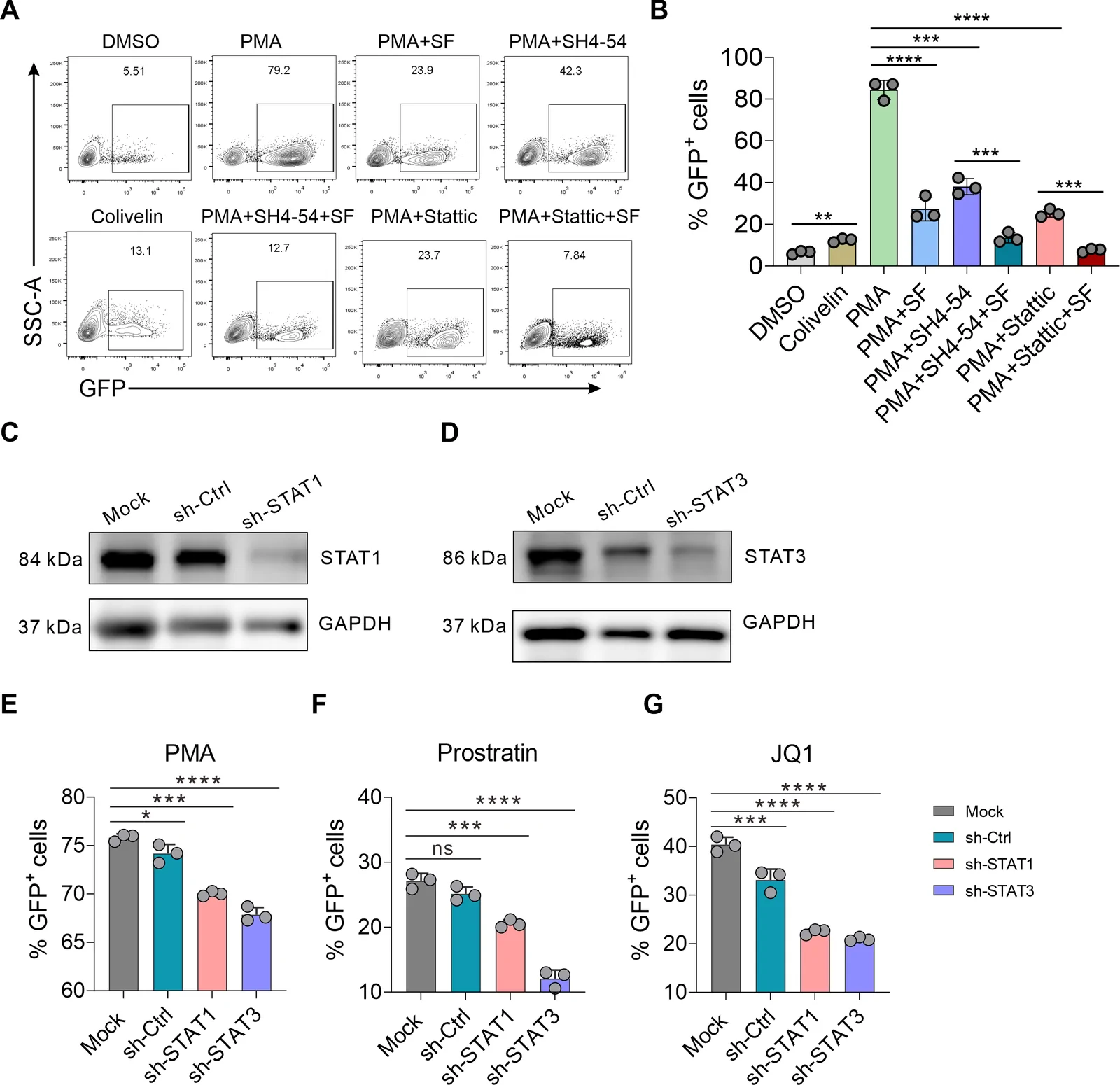
STAT1/3 signaling is required for HIV-1 latency reactivation. (**A and B**) Effects of STAT family protein inhibitors (SH4-54 and stattic) and agonist (colivelin) on reactivation of latent HIV-1. J-Lat A2 cells were treated with DMSO, colivelin (2 μM), PMA (10 ng/mL), PMA + sorafenib (4 μM), PMA + SH4-54 (4 μM), PMA + SH4-54+sorafenib, PMA + stattic (8 μM), or PMA + stattic + sorafenib for 48 h and then collected for fluorescence-activated cell sorting (FACS). Representative flow cytometry plots showing the frequency (**A**) and quantification (**B**) of GFP^+^ cells on day 2 post-treatment. (**C and D**) Efficient knockdown of STAT1 or STAT3 in J-Lat cells was confirmed by western blot analysis. (**E–G**) Genetic silencing of STAT1 or STAT3 attenuates HIV-1 latency reactivation. Control and STAT1- or STAT3-knockdown cells were stimulated with PMA (**E**), prostratin (**F**), or JQ1 (**G**), and HIV-1 reactivation was assessed by GFP expression. Each group of data represents the average of three replicates. All data are means ± SD. *P*-values calculated using unpaired *t*-test, ns, not significant, *, *P*<0.05, **, *P*<0.01, ***, *P*<0.001, and ****, *P*<0.0001. Brackets indicating specific group comparisons are shown on each graph.

To further validate the functional role of STAT1 and STAT3 in HIV-1 latency reactivation, we employed both pharmacological and genetic approaches. Pharmacological inhibition of STAT3 using SH-4-54 and stattic significantly reduced the proportion of GFP^+^ cells following PMA stimulation (Fig. 7A and B). Notably, co-treatment with sorafenib further enhanced the inhibitory effects of SH-4-54 and stattic on PMA-induced HIV-1 reactivation, indicating a cooperative suppression of STAT-dependent transcriptional activation. In contrast, treatment with colivelin, a well-characterized STAT3 activator, markedly increased HIV-1 reactivation when administered alone, as reflected by an increased percentage of GFP^+^ cells (Fig. 7A and B). To further establish the requirement of STAT signaling, we performed knockdown of STAT1 or STAT3 in J-Lat cells (Fig. 7C and D). Silencing of either STAT1 or STAT3 significantly attenuated PKC-mediated reactivation of latent HIV-1, as well as JQ1-induced reactivation (Fig. 7E through G). Collectively, these results demonstrate that STAT1 and STAT3 are critical regulators of HIV-1 transcriptional reactivation and are required for efficient latency reversal across distinct activating stimuli, supporting their role as key downstream effectors in PKC-driven signaling pathways.

## DISCUSSION

**Fig 8:**
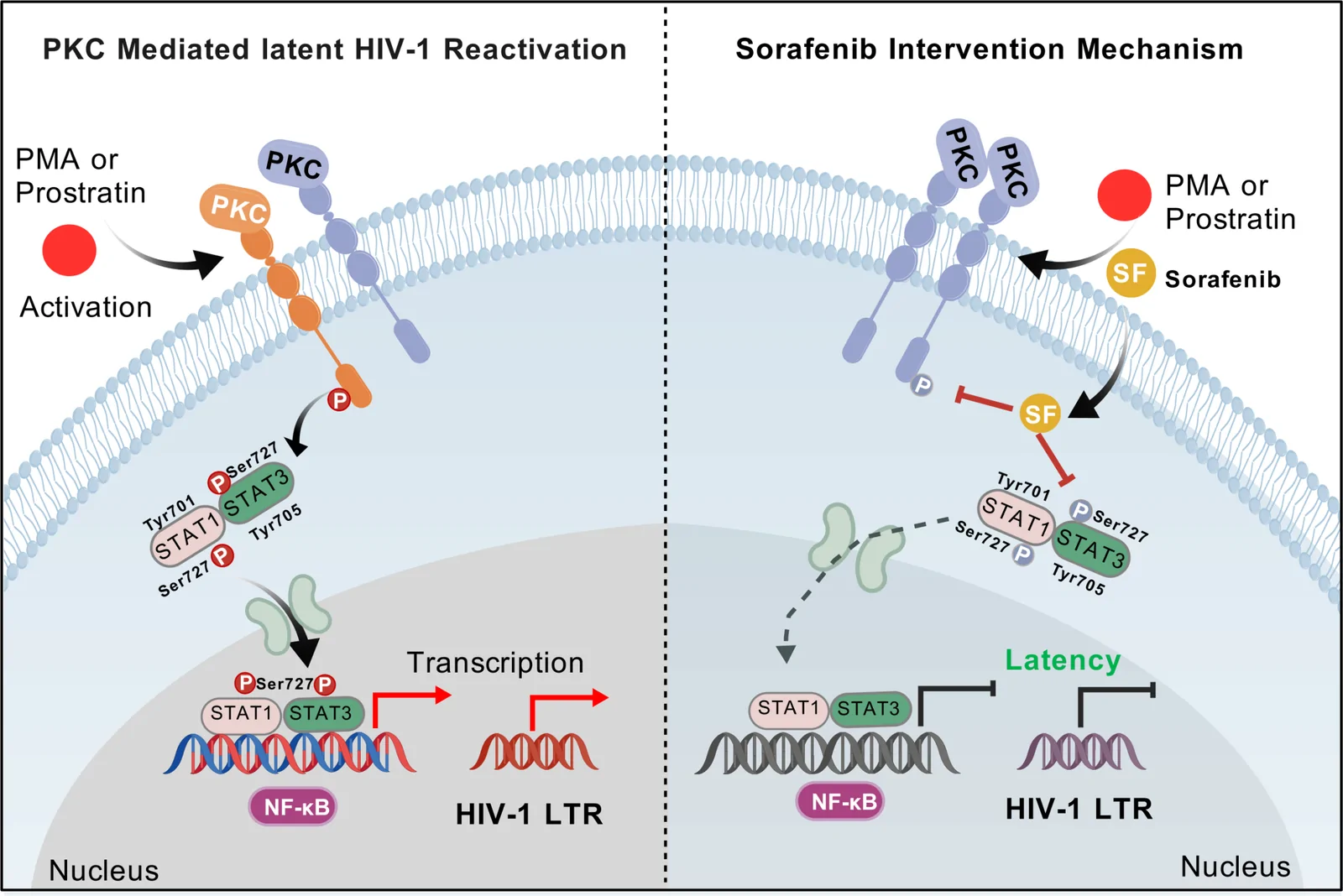
Sorafenib enforces HIV-1 latency by targeting a critical signaling axis that links PKC activation to viral transcription. By selectively inhibiting STAT1/3 serine 727 phosphorylation, sorafenib disrupts transcriptional activation of the HIV-1 LTR without affecting canonical tyrosine phosphorylation. This mechanism uncouples upstream activation signals from viral reactivation, highlighting STAT1/3 as a therapeutic node for “block and lock” strategies toward a functional HIV-1 cure.

The pursuit of an HIV-1 cure remains one of the most formidable challenges in contemporary medicine. Compared to a sterilizing cure, which aims to completely eradicate the virus, a functional cure—defined by long-term viral suppression in the absence of ART—represents a more attainable and pragmatic goal. Among current strategies, the “block and lock” approach has garnered increasing attention as a promising avenue toward durable viral remission. LPAs play a central role in this strategy by enforcing sustained transcriptional silencing of the latent reservoir and preventing viral reactivation (24–26). However, despite encouraging progress, existing LPAs generally exhibit limited potency, incomplete suppression of reactivation, and suboptimal clinical efficacy (27, 41), underscoring the need for safer and more effective compounds. In the present study, we identified sorafenib as a novel LPA candidate through high-throughput screening and demonstrated its ability to suppress HIV-1 reactivation across multiple latency models while promoting latency establishment in primary CD4^+^ T cells. Importantly, we further delineate a mechanistic basis for this effect, showing that sorafenib inhibits PKC-driven signaling and selectively suppresses STAT1/3 serine 727 phosphorylation, thereby impairing transcriptional activation at the HIV-1 LTR (Fig. 8).

Sorafenib (BAY 43-9006, marketed as Nexavar) is a multi-kinase inhibitor with established anti-angiogenic and anti-proliferative properties (45–47). It exerts its therapeutic effects primarily through inhibition of multiple receptor tyrosine kinases, including c-Met, VEGFRs, and PDGFRs (46, 48, 49), as well as components of the Ras/Raf/MEK/ERK signaling cascade (50, 51). The U.S. FDA has approved sorafenib for the treatment of advanced hepatocellular carcinoma and renal cell carcinoma (52–55), highlighting its favorable clinical safety profile and making it an attractive candidate for drug repurposing. Consistent with these observations, our data show that sorafenib exhibits minimal cytotoxicity and does not significantly alter primary CD4^+^ T cell phenotypes, including activation, exhaustion, or effector function, at pharmacologically relevant concentrations. These findings align with previous reports of sorafenib tolerability (56–59), including studies in HIV-1-infected individuals with hepatocellular carcinoma (60, 61). Notably, our work expands the functional scope of sorafenib by demonstrating, for the first time, its capacity to regulate HIV-1 latency, thereby revealing a previously unrecognized antiviral application.

Mechanistically, the JAK-STAT pathway functions as a central signaling hub that transduces extracellular cytokine signals into transcriptional responses (62, 63). Pharmacological inhibition of JAK kinases, such as with tofacitinib and ruxolitinib, has been shown to reduce HIV-1 replication, limit reservoir establishment, and modulate CCR5 expression in CD4^+^ T cells (64, 65). STAT phosphorylation is a critical step in this pathway, with distinct family members exerting diverse effects on HIV-1 biology. While STAT1 and STAT3 are generally associated with antiviral responses (66–69), STAT5 has been shown to directly promote HIV-1 transcription via interactions with the viral LTR (70). In addition, HIV-1 accessory proteins such as Vif can modulate STAT signaling by inhibiting interferon-induced phosphorylation of STAT1/3 (68).

Beyond canonical tyrosine phosphorylation, serine phosphorylation plays a crucial role in regulating the transcriptional activity of STAT proteins (71, 72). Phosphorylation of STAT1 at serine 727 is required for full transcriptional activation (73, 74), and a similar requirement has been described for STAT3, where serine 727 phosphorylation enhances transcriptional output without altering DNA-binding affinity (75). In this study, we demonstrate that sorafenib selectively inhibits serine 727 phosphorylation of STAT1 and STAT3, a modification induced downstream of PKC signaling. By targeting this non-canonical regulatory step, sorafenib likely disrupts the recruitment of transcriptional co-activators and impairs STAT-dependent transcription at the HIV-1 promoter, thereby maintaining proviral silencing. Importantly, this mechanism is distinct from conventional kinase inhibitors that primarily target tyrosine phosphorylation events. Our findings are consistent with emerging evidence implicating serine-directed kinases, including mTOR, CDK8, and CDK9, in the regulation of HIV-1 transcription and latency reversal (76–78). Furthermore, the ability of sorafenib to modulate multiple upstream signaling pathways—including RAF, VEGFR, and PDGFR—may contribute to its capacity to suppress redundant or compensatory pathways that drive HIV-1 reactivation.

Despite these promising findings, several limitations should be acknowledged. First, our study is based primarily on *in vitro* latency models, which may not fully recapitulate the complexity and heterogeneity of the latent reservoir *in vivo*. Second, while sorafenib effectively enforces latency over short timeframes, the long-term durability of this “locked” state remains unclear and requires further investigation. Given that sorafenib primarily functions as a kinase inhibitor, sustained or repeated dosing may be required to maintain long-term suppression of HIV-1 reactivation. However, prolonged inhibition of STAT-mediated transcriptional activation may also promote the gradual establishment of repressive epigenetic modifications at the HIV-1 *LTR*, potentially contributing to a more durable “locked” state that persists after drug withdrawal. Future studies examining the epigenetic status of the proviral *LTR* following extended sorafenib treatment and washout will help clarify this possibility. Third, given the broad kinase inhibition profile of sorafenib, potential off-target effects and their impact on immune function warrant additional study. Future work using *in vivo* systems, including humanized mouse models of HIV-1 latency, will be essential to evaluate the translational potential of sorafenib in the context of HIV-1 cure strategies.

In summary, our study defines a PKC-STAT1/3 serine 727 signaling axis as a key regulator of HIV-1 latency and identifies sorafenib as a clinically relevant compound capable of targeting this pathway. These findings provide both mechanistic insight and a conceptual framework for developing STAT-centered “block and lock” strategies toward a functional cure for HIV-1.

## MATERIALS AND METHODS

### Cell acquisition and culture

J-Lat 6.3, J-Lat 8.4, J-Lat 9.2, J-Lat 10.6, J-Lat 15.4, and J-Lat A2 cells were initially generated in the laboratory of Eric Verdin (The Buck Institute for Research on Aging, Novato, CA, USA) and were gifted by the laboratory of Robert F. Siliciano (Department of Medicine, Johns Hopkins University School of Medicine, Baltimore, MD, USA). J-mC cells were generated as described previously (43). Peripheral blood from HIV-negative healthy volunteers was provided by the Guangzhou Central Blood Station (Guangzhou, Guangdong Province, China). The volunteers were between 18 and 65 years old; CD4^+^ T cell count was between 560 and 1,100 (cells/μL). Peripheral blood mononuclear cells (PBMCs) were separated using Ficoll-Paque density gradient centrifugation (TBD Science, Beijing, China). All J-Lat cell lines and primary CD4^+^ T cells were cultured in basal medium (RPMI 1640; Gibco) supplemented with 1% penicillin-streptomycin and 10% FBS (Gibco).

### Screening

J-Lat A2 cells (1 × 10^5^ cells) were resuspended in 200 μL of medium in round-bottom 96-well plates and treated with different compounds (TargetMol, Cat#L1200) at 10 μM or 5 μM plus PMA (Sigma, USA) in round-bottom 96-well plates. Treatment with PMA (100 ng/mL) was used as the positive control, and that with blank, PBS, and DMSO were used as negative controls. After 48 h, the proportion of GFP^+^ cells was detected by flow cytometry, and the drug that significantly reduced the proportion of GFP^+^ cells activated by PMA was selected as the candidate drug.

### Flow cytometry analysis

GFP and mCherry fluorescence were measured using a BD LSR Fortessa cell analyzer. Surface staining was performed using fluorochrome-conjugated antibodies, including anti-CD25-PE (BioLegend, Cat# 302606), anti-CD69-FITC (BioLegend, Cat# 310903), anti-PD-1-APC (BioLegend, Cat# 329905), anti-CD4-APC-Cy7 (BioLegend, Cat# 317418), anti-CXCR4-PE-Cy7 (BioLegend, Cat# 306514), anti-CCR5-APC (BioLegend, Cat# 359122), anti-LAG-3-APC (BioLegend, Cat# 369212), and anti-TIM-3-PE (BioLegend, Cat# 345006). The cell death ratio was evaluated using the annexin V/PI double-staining assay. Cells were incubated with annexin V-FITC (BioLegend, Cat# 640941) at room temperature for 15 min, and PI (BioLegend, Cat# 421301) was added. The flow cytometry data were acquired using a BD LSR Fortessa instrument. The data were analyzed using FlowJo software.

### Cytotoxicity assays

Cell mortality was determined using annexin V/PI double staining. Unactivated primary CD4^+^ T cells were treated with DMSO, PMA (10 ng/mL) plus ionomycin (1 μM), SAHA (1 μM), or sorafenib (4 μM) for 48 h. Annexin V was incubated at room temperature for 15 min, PI was added, and the stained cells were analyzed by flow cytometry. All reagents were purchased from BioLegend. Fluorescence values were analyzed using FlowJo software.

### Western blotting

After treatment, cells were lysed by radioimmunoprecipitation lysis buffer (10 mM Tris-HCl buffered at pH 7.5, 150 mM NaCl, 0.5% NP-40, 1% Triton X-100, 10% glycerol, 2 mM Ethylenediaminetetraacetic acid, 1 mM NaF, 1 mM Na_3_VO_4_, 1 × protease, and phosphatase inhibitor cocktail) and incubated on ice for 30 min, followed by centrifugation at 12,000 × *g* for 10 min at 4°C. The supernatant was collected as the whole protein extract. Protein samples were stored at −80°C or used directly for western blotting. The protein extract was denatured by adding NuPAGE running buffer (Thermo Fisher Scientific) and heated at 100°C for 10 min. The 50 µg total protein was electrophoresed on a 10% polyacrylamide gel for 1.5 h to isolate the protein, which was then transferred onto a polyvinylidene fluoride membrane (Bio-Rad, Hercules, CA, USA). They were then co-incubated with primary antibodies and incubated with secondary antibodies conjugated to horseradish peroxidase (HRP). Antibodies were diluted with 5% bovine serum albumin. Primary antibodies against AKTs (PK30020, Proteintech), Phospho-mTOR (Ser2448; 67778-1-Ig, Proteintech), mTOR (66888-1-Ig, Proteintech), PI3 Kinase p110 Beta (20584-1-AP, Proteintech), Phospho-STAT1 (Ser727; 28977-1-AP, Proteintech), STAT1 (66545-1-Ig, Proteintech), Phospho-JAK2 (Tyr1007/1008; AF1486, Beyotime), JAK2 (AF1489, Beyotime), Phospho-JAK3 (Tyr980/981) (29101-1-AP, Proteintech), JAK3 (80331-1-RR, Proteintech), STAT3 (10253-2-AP, Proteintech), STAT5A/B (13179-1-AP, Proteintech), and Phospho-STATs (9914T, CST). HRP-labeled Goat Anti-Mouse IgG (H + L; A0216) and HRP-labeled Goat Anti-Rabbit IgG (H + L; A0208) were purchased from Beyotime (Guangzhou, China). The HRP signal was detected using ChemiDoc Touch (Bio-Rad). Densitometric quantification of western blot bands was performed using ImageJ software. Band intensities were normalized to the corresponding loading control (GAPDH), and phosphoprotein/total protein ratios were calculated from at least three independent experiments.

### RNA-seq

J-Lat A2 cells were treated with DMSO (Sigma-Aldrich Cat# D2650), PMA (10 ng/mL), sorafenib (4 μM, TargetMol, Cat#T0093L), and PMA (10 ng/mL) plus sorafenib (4 μM) for 48 h. Total RNA from each group was extracted using TRIzol reagent and quantified using a Nanodrop 2000 spectrophotometer (Thermo Fisher). RNA quality was determined using an Agilent 2100 Bioanalyzer. The RNA-Seq library was built using a TruSeq stranded mRNA library prep kit (Illumina) and sequenced using the HiSeq X Ten platform (Illumina) at Novogene (Beijing, China). Differential expression analysis was performed using the DEGseq R package (version 1.48.0). As RNA-seq was conducted without biological replicates, the MA-plot-based method with the Random Sampling model implemented in DEGseq was used for identifying differentially expressed genes (DEGs), which estimates variance from the data distribution without requiring biological replicates. DEGs were identified based on an absolute log2 fold change ≥1 and a *q*-value (Benjamini-Hochberg adjusted *P*-value) ≤0.05. We acknowledge that the absence of biological replicates is a limitation of the RNA-seq analysis; accordingly, transcriptomic findings were validated through independent biochemical experiments (western blot analysis of signaling pathway components).

### Virus stock production

Pseudotyped viral stocks were produced in HEK293 T cells by co-transfecting 2.2 μg of pCXCR4 HIV-1 envelope-expression vector, 4.4 μg of packaging vector pC-Help, and 4.4 μg pDFV-B construct using a polyethylenimine (Invitrogen) transfection system according to the manufacturer’s instructions. Supernatants were harvested after 48 h, centrifuged (10 min, 500 × *g*, room temperature), and filtered through a 0.45 μm pore size membrane to remove the cell debris. Viruses were concentrated by centrifuging with a 25% volume of 50% polyethylene glycol 6000 and a 10% volume of 4 M NaCl. Concentrated virions were resuspended in complete medium and stored at −80°C.

### Infection of primary CD4^+^ T cells

Primary CD4^+^ T cells were isolated from HIV-naïve individuals. CD4^+^ T cells were co-stimulated with anti-CD3 and anti-CD28 antibodies for 3 days. Activated CD4^+^ T cells were then infected with DFV-B incorporating a CXCR4-tropic Env at an MOI of 0.05–0.1. The infected cells were maintained in super T cell medium for 3 days after infection, then the medium was changed to cell-free medium, and cultured for 1 week. For delayed treatment experiments, sorafenib (4 μM) was added on day 3 post-infection and maintained continuously in the culture medium until the analysis endpoint at day 7 post-infection.

### Statistical analysis

All experiments were repeated at least three times. For cell line-based experiments (e.g., J-Lat models), representative data from one of at least three independent experiments are shown, with error bars representing three technical replicates within that experiment. For primary CD4^+^ T cell experiments, data represent biological replicates from independent donors. Statistics were performed with GraphPad Prism 8 (GraphPad Software, Inc., San Diego, CA, USA), and all data are presented as mean values ± SD. *P*-values were calculated based on the Student’s *t*-test using IBM SPSS Statistics for Windows, version 20.0 (IBM Corp., Armonk, NY, USA). * Denotes *P* < 0.05, ** denotes *P* < 0.01, *** denotes *P* < 0.001, **** denotes *P* < 0.0001, and ns denotes non-significant.

## SUPPLEMENTAL MATERIAL

Table S1 aac.00540-26-s0001.docx Summary of proviral integration sites in latent HIV-1 J-lat cell line models.

ASM does not own the copyrights to Supplemental Material that may be linked to, or accessed through, an article. The authors have granted ASM a non-exclusive, world-wide license to publish the Supplemental Material files. Please contact the corresponding author directly for reuse.
