# Supplementary material for: Sorafenib enforces HIV-1 latency by selectively inhibiting PKC-driven STAT1/3 serine 727 phosphorylation
Source: Antimicrob Agents Chemother. 2026 Aug 3;70(9):e00540-26. doi: 10.1128/aac.00540-26 (PMC13712939; doi:10.1128/aac.00540-26)
Supplement: Supplementary file 1 [file aac.00540-26-s0001.docx]

**Supplementary Table 1. Summary of Proviral Integration Sites in Latent HIV-1 J-Lat Cell Line Models.**

| **Cell line** | **Parental cell line** | **Integrated gene** | **Chromosome site** |
| --- | --- | --- | --- |
| J-Lat 6.3 | Jurkat | PPP5C | Chromosome 19 |
| J-Lat 8.4 | Jurkat | FUBP1 | Chromosome 1 |
| J-Lat 9.2 | Jurkat | PPP5C | Chromosome 19 |
| J-Lat 10.6 | Jurkat | SEC16A | Chromosome 9 |
| J-Lat 15.4 | Jurkat | UBA2 | Chromosome 19 |
